# Metasurface of Combined Semicircular Rings with Orthogonal Slit Pairs for Generation of Dual Vector Beams

**DOI:** 10.3390/nano11071718

**Published:** 2021-06-29

**Authors:** Qian Kong, Manna Gu, Xiangyu Zeng, Rui Sun, Yuqin Zhang, Chunxiang Liu, Hong Ma, Weiling Gui, Chuanfu Cheng

**Affiliations:** College of Physics and Electronics, Shandong Normal University, Jinan 250014, China; kongqian0304@163.com (Q.K.); gumanna1996@outlook.com (M.G.); zengxiangyu0611@163.com (X.Z.); sunrui199812@163.com (R.S.); ss_yghg@163.com (Y.Z.); liuchunxiang@sdnu.edu.cn (C.L.); mahong@sdnu.edu.cn (H.M.)

**Keywords:** metasurface, dual vector beams, semicircular rings, orthogonal slit pairs, geometric phase

## Abstract

Manipulation of multichannel vector beams (VBs) with metasurfaces is an important topic and holds potential applications in information technology. In this paper, we propose a novel metasurface for the generation of dual VBs, which is composed of orthogonal slit pairs arranged on multiple groups of combined semicircular rings (CSRs). A group of CSRs include a right-shifted set and a left-shifted set of semicircular rings, and each set of semicircular rings has two halves of circles with different radii, sharing the same shifted center. Under the illumination of linearly polarized light, the two shifted sets of semicircular rings generate the two VBs at the shifted center positions on the observation plane. The slit units of each set are designed with independent rotation order and initial orientation angle. By adjusting the linear polarization of illumination, both two VBs with their orders and polarization states are independently controlled simultaneously. The principle and design are demonstrated by the finite-difference time domain (FDTD) simulation. The work is of significance for miniatured devices of VB generators and for related applications.

## 1. Introduction

Due to their peculiar characteristics, vector fields of light have attracted extensive research interest [1,2], and have found versatile, important applications in past decades. Fundamentally, vector beams (VBs) are the superpositions of the total angular momentum (TAM) modes that couple the spin angular momentum (SAM) and orbital angular momentum (OAM), and they can be geometrically described by the high-order Poincaré (HOP) spheres [3,4,5]. Experimentally, various methods have been proposed to generate VBs by manipulating the eigenstates of the TAM. Using different optical devices such as wave plate [6], Daman grating [7], and q-plate [8] in different optical systems of Michelson [9], Mach-Zehnder [10], and Sagnac interferometers, the generation of a variety of single and arrayed high-order VBs has been realized. VBs have been applied in important classical areas, such as optical trapping [11], laser structuring [12], and high resolution microscopy [13]. In quantum science, VBs provide novel resources for quantum information protocol [14] and have enabled quantum applications such as quantum key distribution [15] and quantum walks [16].

Metasurfaces are inhomogeneous and anisotropic surfaces of artificially arrayed sub-wavelength structures of metal or dielectric medium, which make use of interactions of light with the structures to control the amplitude, phase, and polarization of the output light waves. They have found applications in various fields such as anomalous refraction [17], the second harmonic generation [18], and spatial phase control [19,20]. The manipulation of vector light fields with metasurfaces is one of most important subjects in micro-nano photonics. In earlier studies, the different single metal nanoslits were used to generate the radially polarized VB [21,22], and afterwards metasurfaces were developed to construct gradient geometric phases for the generation of more general VBs [23,24,25]. In recent years, rapid advances have been achieved in the generation of high-order and arbitrary VBs with metasurfaces, including the generation of HOP beams [26,27], hybrid order Poincaré beams [28], nondiffracting VBs [29], and broadband VBs [30]. With promising applications in information technology, multiplexing metasurfaces for simultaneously achieving multiple VBs have garnered particular interest and important achievements have been made rapidly. With polarization-controlled superpositions of OAM states, the generation of multi-channel HOP beams are realized [31]. With the metalens phase design based on polarization control, multi-channel focused VBs are generated [32,33], and with a single noninterleaved metasurface, the HOP beams of high capacity are performed successfully [34].

In our present work, we propose a novel metasurface with units of orthogonal slit pairs arranged on multiple groups of combined semicircular rings (CSRs) to generate the tightly focused dual VBs. The generation of dual VBs is of fundamental significance for the generation of multi-channel VBs, and they have found extensive applications in information coding and transmissions [35], information security [36] and spin-decoupled metasurface devices [37]. A group of the combined rings includes a right-shifted set and a left-shifted set. Each set of semicircular rings has two halves of circles sharing the same center, but one with a larger and the other a smaller radius, and each semicircle is the Fresnel zone with optical path differences of integer multiples of wavelength. For a unit of the slit pair, the primary slit is located on these rings, the attached slit is located at the position with path difference of a half-wavelength, and the unit converts the incident circular polarization into opposite circular polarization for output field. The orientations of the units in the metasurface on a set of shifted rings rotate with azimuthal angle, which provides a helical geometric phase for formation of optical vortex and generation of shifted VB. With the units are arranged on the two sets of rings in the metasurface, and with illumination of linearly polarized light, the right-shifted and left-shifted VBs are achieved, and the generation of the dual VB is realized. The slit units on each set of semicircular rings are designed with independent rotation order and initial orientation angle, and in combination with the linear polarization of illumination, both the right- and left-shifted VBs with the orders and polarization states are independently controlled simultaneously. The principle and design are validated by the simulation of finite-difference time domain (FDTD) method. This work is of significance for broadening the applications of metasurfaces, for device miniaturization of VB generators, and for applications in information technology.

This paper is organized as follows. In Section 2, we theoretically analyze the transmitted light field of the slit pair unit (Section 2.1), and derive the Bessel beams produced by the metasurface with slit pairs on a single circular ring under circularly polarized illumination (Section 2.2), and the VB under linear polarization illumination (Section 2.3); we also design of the metasurface with a group combined rings and demonstrate the right- and left-shifted dual VBs. In Section 3, we design the specific metasurface samples and performed the FDTD simulations of dual VBs of radial, 135°-slanted, azimuthal, and 45°-slanted polarizations, and dual beams of radial and azimuthal polarization of first and second orders. In Section 4 and Section 5, discussions and conclusions are presented.

## 2. Materials and Methods

### 2.1. The Unit of the Orthogonal Slit Pair and the Transmitted Light Field

**Figure 1:**
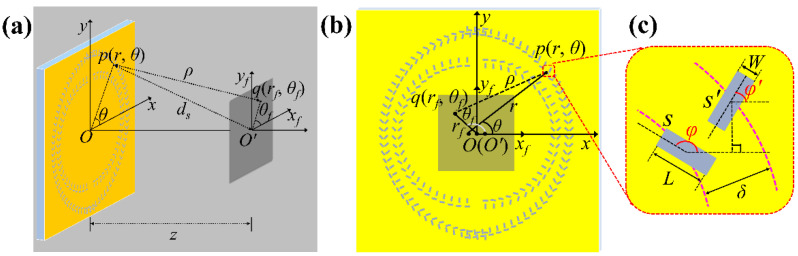
(**a**) Geometry of the optical system for generating dual VBs with metasurface sample of CSRs, z = 10 μm. (**b**) Overlapped view of the sample plane *Oxy* and the observation plane *O’x_f_y_f_* for geometrical analysis. (**c**) Enlarged view of a unit of slit pair. The length (*L*) and width (*W*) of a single slit element are *L* = 300 nm and *W* = 100 nm, respectively; optical path difference *δ* between from the slits s and s’ to *O*’ is *λ**/***2.

Figure 1a is the schematic of the optical system to produce the separated double vortices and dual VBs with the metasurface of CSRs with orthogonal slit pairs etched on gold film. A plane wave light of He-Ne laser with wavelength *λ* = 632.8 nm illuminates the metasurface sample S, which lies in the object plane *Oxy*, and the observation plane is *O’x_f_ y_f_* with the distance *z* from the object plane. Figure 1b demonstrates the overlapped view for the two planes of *Oxy* and *O’x_f_ y_f_*. A slit element *s* of the metasurface sample is located at point *p*(*r*, *θ*), and the distance from *p*(*r*, *θ*) to a point *q* (*r_f_*, *θ_f_*) on the observation plane is *ρ*. The orientation angle of element *s* with respect to *x-*axis is *φ*(*θ*), and the optical path difference between a slit at *p*(*r*, *θ*) and the origin *O’* of the observation plane is an integer multiple of wavelength, i.e., *d_s_*-*z* = *nλ*, and the position of the slit satisfies $\sqrt{r_{n}{}^{2}+z^{2}}-z=n\lambda$ with *n* = 0, 1, 2, ... , indicating that the slit is on the circular ring of the wave zone with the path difference of wavelengths of integer *n*.

Our metasurface is designed to be composed of the building unit of orthogonal slit pairs, and slit *s’* attaching to *s* is perpendicular to *s*, and *s’* is located on an outer to *s* with path difference *λ**/***2 to *O’.* We first consider the light field transmitted through the unit of a slit pair when the light of illumination is circularly polarized, i.e., $E^{\sigma }=({\hat{e}}_{x}+i\sigma {\hat{e}}_{y})/\sqrt{2}$, where *σ* = +1 and *σ* = −1 represent left-handed and right-handed circularly polarized light, respectively. It is well understood that the polarization direction of the light wave excited by a narrow slit is perpendicular to the slit, and hence based on the geometric relation, the radial and azimuthal components of the light field passing through the slit *s* can be written as:

(1) $$E_{r}^{\left(s\right)}(r,\theta )=\frac{i\sigma }{2}\exp(i\sigma \varphi )\sin(\theta -\varphi ){\hat{e}}_{r}$$

(2) $$E_{\theta }^{\left(s\right)}(r,\theta )=\frac{i\sigma }{2}\exp(i\sigma \varphi )\cos(\theta -\varphi ){\hat{e}}_{\theta }$$

Similarly, the two components of the transmitted light field of the attaching slit *s’* can also be obtained, merely by substituting *φ’* = *φ* − *π*/2 for *φ* in the above equation; and noticing the phase difference of *π* due to the path difference of *λ/*2, the transmitted light field of the slit pair of *s* and *s’* can be calculated as:

(3) $$\begin{array}{l} E(r,\theta )=E^{\left(s\right)}+E^{\left(s\prime \right)}\exp(i\pi )\\ =\frac{1}{\sqrt{2}}\exp[i\sigma (2\varphi \prime -\theta )]{\hat{e}}_{r}-\frac{i\sigma }{\sqrt{2}}\exp[i\sigma (2\varphi \prime -\theta )]{\hat{e}}_{\theta }\\ =\frac{1}{\sqrt{2}}\exp\{i\sigma [2(\varphi -\frac{\pi }{2})-\theta ]\}{\hat{e}}_{r}-\frac{i\sigma }{\sqrt{2}}\exp\{i\sigma [2(\varphi -\frac{\pi }{2})-\theta ]\}{\hat{e}}_{\theta }\\ =E_{r}(r,\theta ){\hat{e}}_{r}-E_{\theta }(r,\theta ){\hat{e}}_{\theta } \end{array}$$

With the rotation transformation of coordinate systems, the above expression can be written in *x*- and *y*- components:

(4) $$E(r,\theta )=\left[\begin{array}{l} E_{x}(r,\theta )\\ E_{y}(r,\theta ) \end{array}\right]=\left[\begin{array}{l} \cos\theta \;-\sin\theta \\ \sin\theta \;\cos\theta \end{array}\right]\;\left[\begin{array}{l} E_{r}(r,\theta )\\ E_{\theta }(r,\theta ) \end{array}\right]$$

Using Equation (3), and with simple calculations, it is derived:

(5) $$E(r,\theta )=\left[\begin{array}{l} E_{x}(r,\theta )\\ E_{y}(r,\theta ) \end{array}\right]=\frac{1}{\sqrt{2}}\exp[2i\sigma (\varphi -\frac{\pi }{2})]\left[\begin{array}{c} 1\\ -\sigma i \end{array}\right]$$

The above equation indicates that the slit pair of *s’* and *s* transforms the incident light of circular polarization (CP) [1 *iσ*]*^T^* into the outgoing field of opposite CP [1 − *iσ*]*^T^* with the geometric phase $\exp[2i\sigma (\varphi -\frac{\pi }{2})]$ imposed. This means that a pair of slits acts equivalently as a half-wave plate with the direction of the fast axis at the angle of *ϕ* − *π/*2 with respect to the *x*-axis. In the following analysis, we use the pair of slits as a unit, and specify it as slit*s* unless otherwise noted.

### 2.2. The Output Light Field of the Slit Pairs on a Circular Ring under Circular Polarization Illumination

We now consider the metasurface consisting of the slits arranged on single circular ring. For slits at the position *p*(*r*, *θ*) with *r* the radius of the ring, the area element is expressed as d*A* = *r*d*r*d*θ*, and *r*d*θ =* d*l* and d*r* are the length and the width, respectively, of d*A*. According to the Huygens–Fresnel principle of surface plasmons [38], the light field $U_{1}(r_{f},\theta_{f})$ at point *q*(*r_f_*, *θ_f_*) is the superposition of all slit units on the ring on the observation plane and it can be written as the following integral [38]:

(6) $$\begin{array}{ll} U_{1}(r_{f},\theta_{f}) & =\frac{-i}{\sqrt{\rho \lambda }}\int E(r,\theta )\exp[i(k\rho +\frac{\pi }{4})]rdrd\theta \\ & =\frac{-i}{\sqrt{\rho \lambda }}\int \exp[2i\sigma (\varphi -\frac{\pi }{2})]\exp[i(k\rho +\frac{\pi }{4})]\left[\begin{array}{c} 1\\ -\sigma i \end{array}\right]rdrd\theta \end{array}$$

where

(7) $$\rho \approx {\left(z^{2}+r^{2}\right)}^{1/2}+[r_{f}{}^{2}-2r_{f}r\cos(\theta -\theta_{f})]/2{\left(z^{2}+r^{2}\right)}^{1/2}$$

In the area near the center of the observation plane, $r_{f}{}^{2}$ can be ignored, and the term in the denominator $1/(z^{2}+r^{2}{)}^{1/2}$ can be approximated as 1/z. The above equation is written as:

(8) $$\rho \approx {\left(z^{2}+r^{2}\right)}^{1/2}-2r_{f}r\cos(\theta -\theta_{f})/2z$$

Substituting the above equation and Equation (5) into Equation (6), we obtain:

(9) $$U_{1}(r_{f},\theta_{f})=\frac{-i}{\sqrt{\rho \lambda }}\int \exp[2i\sigma (\varphi -\frac{\pi }{2})]\exp\{ik[(z^{2}+r^{2}{)}^{1/2}-2r_{f}r\cos(\theta -\theta_{f})/2z]\}\left[\begin{array}{c} 1\\ -\sigma i \end{array}\right]rdrd\theta$$

To generate the light field of an optical vortex, we consider the slits rotating on the ring with the orientation varying azimuthally according to the following relation:

(10) $$\varphi =m\theta +\varphi_{0}$$

where *m* is called the rotation order, *φ*_0_ is the orientation angle of the slit at *θ* = 0, and is referred to as the initial orientation angle. Substituting the above into Equation (9), and considering that the ring is on the Fresnel zone with the position satisfying *k*(*z*^2^ + *r*^2^)^1*/*2^ *=* 2 *nπ*, we have:

(11) $$U_{1}(r_{f},\theta_{f})=\frac{-i{\left(-i\right)}^{l}}{\sqrt{\rho \lambda }}\exp[2i\sigma (-\frac{\pi }{2}+\varphi_{0})]\exp(il\theta_{f})J_{l}(ar_{f})\left[\begin{array}{c} 1\\ -i\sigma \end{array}\right]$$

where *l =* 2*σm*, *a = kr_n/_z*. The above equation shows that the light field generated by the metasurface of slits rotating on the Fresnel zone ring is a Bessel vortex beam of CP opposite to the incident light. In particular, when *l* = 0, i.e., *m* = 0, the slits do not rotate on the ring, and a central bright spot of *J*_0_(*ar_f_*) will be generated; while *m* = 1*/*2, i.e., *l* = 1, the first-order Bessel vortex beam with topological charge 1 will be generated.

### 2.3. The Vector Beams Produced by Slit Pairs on an Circular Ring under Illumination of Linear Polarized Light

When the light of horizontal linear polarization *E^h^* is used for illumination, it is written as the addition of two CP waves:

(12) $$E^{h}=E^{\sigma (1)}+E^{\sigma (-1)}=\frac{1}{\sqrt{2}}\left[\begin{array}{l} \;1\\ \;i \end{array}\right]+\frac{1}{\sqrt{2}}\left[\begin{array}{l} \;1\\ -i \end{array}\right]$$

where *E^σ^*^(1)^ and *E^σ^*^(−1)^ represent left CP (LCP) and right CP (RCP) with *σ* = 1 and *σ* = −1 respectively. Correspondingly, the light field *U_h_*_1_(*r_f_*, *θ_f_*) on the observation plane is the addition of the light fields produced by the two illuminating CPs. Based on Equations (11) and (12), and using *l* = 2 *σm*, we have:

(13) $$\begin{array}{ll} U_{h1}(r_{f},\theta_{f}) & =\frac{-i}{\sqrt{\lambda \rho }}\{(+i{)}^{2m}\exp[2i(-\frac{\pi }{2}+\varphi_{0})]\exp(i2m\theta_{f})J_{2m}(ar_{f})\left[\begin{array}{l} \;1\\ -i \end{array}\right]\\ & +(+i{)}^{-2m}\exp[-2i(-\frac{\pi }{2}+\varphi_{0})]\exp(-i2m\theta_{f})J_{-2m}(ar_{f})\left[\begin{array}{l} \;1\;\\ \;i \end{array}\right]\} \end{array}$$

By simplifying the above equation further, we have:

(14) $$\begin{array}{ll} U_{h1}(r_{f},\theta_{f}) & =\frac{-i}{\sqrt{\lambda \rho }}J_{2m}(ar_{f})\{\exp[i(m\pi -\pi +2\varphi_{0})]\exp(i2m\theta_{f})\left[\begin{array}{l} \;1\\ -i \end{array}\right]\\ & +(-1{)}^{2m}\exp[-i(m\pi -\pi +2\varphi_{0})]\exp(-i2m\theta_{f})\left[\begin{array}{l} \;1\\ \;i \end{array}\right]\} \end{array}$$

The above field is superposition of the two opposite vortices of opposite CPs, and it is the VB of order 2*m*. In particular, when *m =* 1*/*2, the above equation is reduced to:

(15) $$U_{h1}(r_{f},\theta_{f})=\frac{1}{\sqrt{\lambda \rho }}J_{1}(ar_{f})\{\exp(-i2\varphi_{0})\exp(-i\theta_{f})\left[\begin{array}{l} \;1\;\\ \;i \end{array}\right]+\exp(i2\varphi_{0})\exp(i\theta_{f})\left[\begin{array}{l} \;1\;\\ -i \end{array}\right]\}$$

which is the VB for order 1. It is noted that the centers of either the vortex beam expressed in Equation (11) or the VB in Equation (15) are at the optical axis, i.e., point *O’* on the observation plane.

### 2.4. The Dual Shifted Spots and Vector Beams Produced by Slit Pairs Arranged on the Combined Semicircular Rings

We now demonstrate how to shift the centers of the vortex beam given in Equation (11) and the VB in Equation (15) by using the CSRs of Fresnel zones, and how to generate the separated dual vortices and VBs.

**Figure 2:**
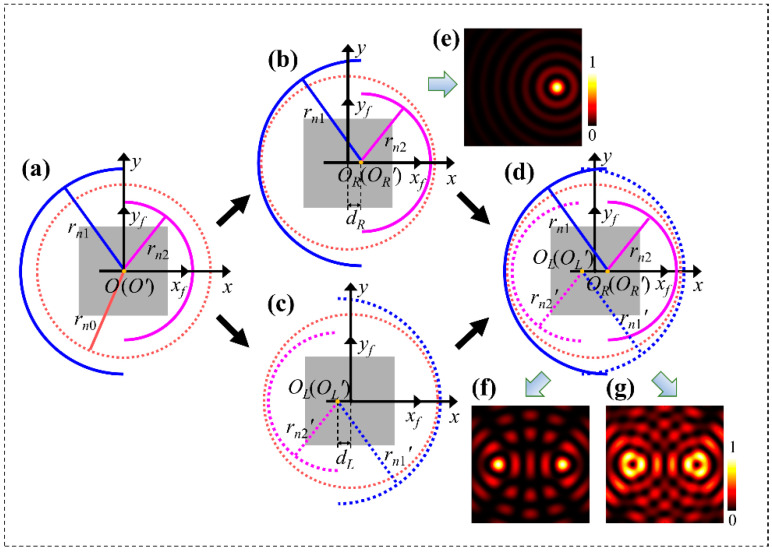
Schematic for the CSRs of Fresnel zone. (**a**) The two semicircular rings of *n*_2_-th and *n*_1_-th Fresnel zones with the same center *O*. (**b**) The right-shifted set of semicircular rings with the center shifted to *O_R_*. (**c**) The left-shifted set of semicircular rings with the center shifted to *O_L_*. (**d**) CSRs as the superimposition of the sets in (**b**,**c**). (**e**) The theoretical intensity distribution with the left-shifted spot produced by the structure in (**b**). (**f**,**g**) The theoretical intensity distribution of the dual spots and vortices produced by the metasurface sample shown in (**d**), respectively.

Based on Equation (11), to obtain two light spots shifting from the center of the optical axis under a CP illumination, but to avoid the cross of corresponding circular rings on which the slits are arranged to produce the two spots, we designed two semicircular rings of Fresnel zone with radii of certain difference to replace one complete ring. The principle is schematically shown in Figure 2. We first take the circular ring of the *n*_0_-th Fresnel zone with radius *r_n_*_0_ and center at *O* as the reference, and the optical axis of the system passes through *O*. This ring is shown by the dashed circle in wine-red in Figure 2a. Then, we introduce a set of two semicircular rings of *n*_2_-th and *n*_1_-th Fresnel zones with the same center *O*, while the left and right halves have the radii of *r_n_*_2_ and *r_n_*_1_, respectively, where *n*_2_ > *n*_0_ > *n*_1_, and they are plotted in blue and red solid curves, respectively, also demonstrated in Figure 2a. The semicircular rings as the combined ring are shifted right with a distance *d_R_*, which satisfies *d_R_* ≤ *r_n_*_2_ *− r_n_*_1_, with their common center moved to *O_R_*, as demonstrated in Figure 2b. This set of semicircular rings is specified as the right set. When the slit pairs are arranged on the set of the two semicircular rings and their orientation angles remain unchanged, the geometric phases of the light waves from all the slits will not be varied, and the waves are in phase at the center *O_R_*, leading to the constructive interference to form Bessel beam of central spot at *O_R_*. This means that the beam has also shifted by the distance *d_R_* along with the two semicircular rings. Figure 2e shows the light intensity distribution calculated by the equivalent scalar diffraction of light based on Equation (11), and it can be obviously seen that the light spot undergoes a shift from the point *O’* on the optical axis *OO’*.

By superimposing the two sets of semicircular rings in Figure 2b,c, we obtain the structure of semicircular rings based the Fresnel zones with ring radii *r_n_*_2_, *r_n_*_1_ and *r_n_*_2_*’* = *r_n_*_2_, *r_n_*_1_*’* = *r_n_*_1_, as demonstrated in Figure 2d, and we define the two sets of semicircular rings as a group of CSRs. Here, we further clarify that the solid curves are for the semicircular rings moving to the right, and the dotted curves are for those moving to the left, while the curves in blue are for rings of the larger zone integer *n*_2_ *= n*_2_*’* and in red are for rings of the smaller zone integer *n*_1_ *= n*_1_*’*. Hereafter, we also use the integer *n* to specify the corresponding ring of Fresnel zone for terseness. Furthermore, each semicircular ring represents the orthogonal slit pair with optical path difference of half wavelength. Figure 2f shows theoretical intensity pattern calculated with Equation (11) for the two spots of the zeroth Bessel beams produced by the metasurface of combined rings. Specifically, when LCP light with the helicity or SAM *σ* = 1 is used for illumination, and the rotation order of the slit pairs on the two sets of semicircular rings with *n*_2_, *n*_1_ and *n*_2_*’*, *n*_1_*’* are *m_R_* = *m_L_* = 1/2, two circularly polarized vortices of RCP with helicity −1 and with topological charges *l_R_* = *l_L_* = 1 are formed with the centers at points *O_L_* and *O_R_* with their separation distance of *d_L_ + d_R_*. Figure 2g shows the theoretical light intensity distributions calculated based on Equation (11).

When linearly polarized light is used for illumination, the slit pairs with the rotation order of *m_R_* on the right set will produce the RCP vortex of topological charge *l_R, σ =_*_1_ *=* 2*σm_R_ =* 2*m_R_* by the LCP component of *σ* = 1 in incident light, and simultaneously produce the LCP vortex of topological charge *l_R, σ =_*_−1_ *=* 2*σm_R_ =* −2*m_R_* by the RCP component of *σ* = −1. The superposition of the two circularly polarized vortices form VB of order 2*m_R_* at *O_R_*. In the same way, the slit pairs with the rotation order of *m_L_* on left set will produce VB of order 2*m_L_* at *O_L_*. Mathematically, the VB *U_h_*_1_(*r_f_*, *θ_f_*) with center at *O’* on the observation plane given in Equation (15) is denoted as *U_h_*_1_(*O’*), i.e., *U_h_*_1_(*O’*) = *U_h_*_1_(*r_f_*, *θ_f_*), the VB formed by the right set of semicircular rings *n*_2_ and *n*_1_ is denoted as *U_h_*_1*R*_(*O_R_’*, *n*_2_, *n*_1_*’*) with the center shifted right to *O_R_’*, and the VB by left set of rings *n*_2_*’* and *n*_1_*’* as *U_h_*_1*L*_(*O_L_’*, *n*_2_*’*, *n*_1_*’*) at left to *O_L_’*. Then, the light field generated by the group of the two sets of combined rings can be written as:

(16) $$U_{h1c}=U_{h1R}(O_{R}{}^{\prime },\;n_{2},\;n_{1})+U_{h1L}(O_{L}{}^{\prime },\;n_{2}{}^{\prime },\;n_{1}{}^{\prime })$$

The two VBs on the right side of the above equation have the same form as the beam *U_h_*_1_(*O’*) = *U_h_*_1_(*r_f_*, *θ_f_*) in Equation (15), except that the center points of the two beams move to *O_R_’* and *O_L_’*, respectively.

## 3. Results

### 3.1. The FDTD Results for Metasurface of A Single Group of Combined Semicircular Rings

**Figure 3:**
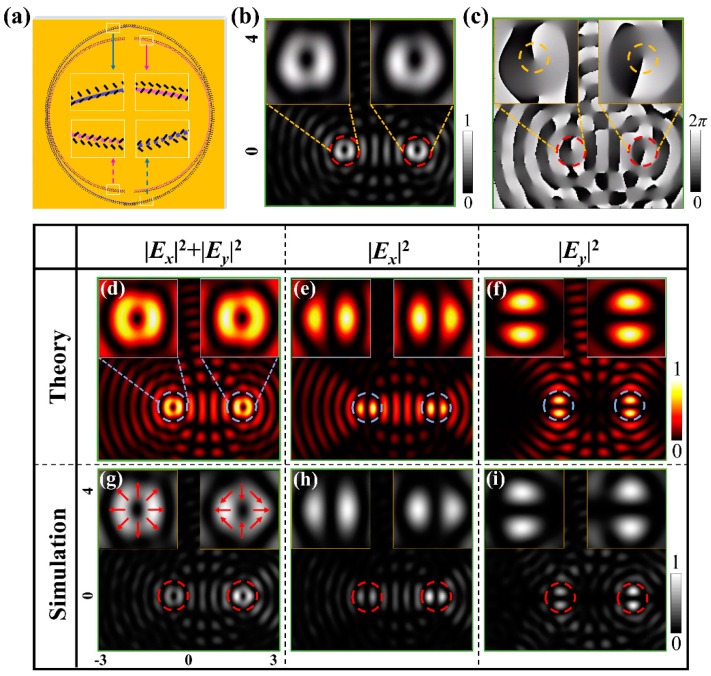
Theory and simulation results of the dual vortices and VBs. (**a**) The metasurface sample of a single group of CSRs with *n*_2_ = *n*_2_*’* = 19 and *n*_1_ = *n*_1_*’* = 15. The FDTD intensity pattern (**b**) and the phase map (**c**) of the dual vortices under illumination of LCP, respectively. (**d**–**f**) Theoretical intensity patterns of *I* = |*E_x_*|^2^ + |*E_y_*|^2^, |*E_x_*|^2^ and |*E_y_*|^2^ of the dual radially polarized VBs. (**g**–**i**) The intensity patterns of FDTD simulations. The insets are the enlarged views. The overlaid arrows show the schematic polarization states.

Based on the principle depicted above, we first design the metasurface containing a group of semicircular rings in the two sets with *n*_2_ = *n*_2_*’* = 19 and *n*_1_ = *n*_1_*’* = 15 and with the corresponding radii of 19.62 μm and 16.73 μm, respectively, as shown in Figure 3a. Each semicircular ring of slit *s* is accompanied by the attaching ring of slit *s’*. The rotational orders of the sample are *m_R_* = −1/2 and *m_L_* = 1/2, and the initial orientation angles for the right and left sets are *φ_R_*_0_ *= φ_L_*_0_ *=* 0°. The method of FDTD is used to simulate the light field produced by the metasurface. In the performance, the nanoslits are modeled to be etched on the metal film of gold with thickness 200 nm, and the substrate is fused silica. The wavelength illuminating light is set as 632.8 nm, i.e., the wavelength of He-Ne laser. The parameters are the same as given in the beginning of the previous section. The length (*L*) and width (*W*) of *L* = 300 nm and *W* = 100 nm are optimized carefully to obtain VBs of good quality. Figure 3b,c show results of the intensity and the phase for the two dual vortices of RCP under illumination of LCP. While under illumination of linear polarization, the two separated radially polarized VBs of order *l_R_* = −1 and *l_L_* = 1 at *O_R_’* and *O_L_’* are obtained, and Figure 3d–f show the theoretical patterns of total intensity *I* = |*E_x_*|^2^ +|*E_y_*|^2^, the component intensities |*E_x_*|^2^ and |*E_y_*|^2^, respectively. Figure 3g–i show the intensity patterns of FDTD simulations, and overlaid arrows in Figure 3g show the schematic polarization states of the beams.

### 3.2. Metasurfaces of Multiple Groups of Semicircular Rings and the FDTD Results for Different Dual Vector Beam

**Figure 4:**
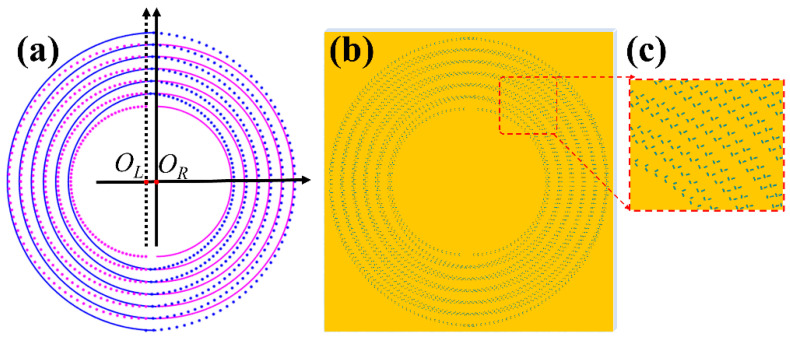

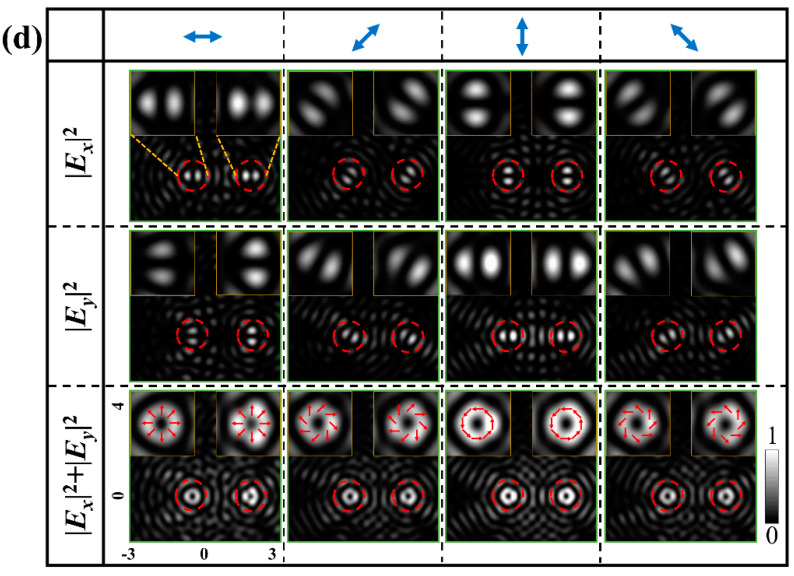
(**a**) Plot of the CSRs. (**b**) The metasurface sample. (**c**) The enlarged view. (**d**) Intensity patterns of the different dual VBs. From left to right columns are dual VBs of the radial, 135°-slanted, azimuthal, and 45°-slanted polarizations produced by sample under the illumination of horizontal, 45°, vertical, and 135° linearly polarized light, respectively. From top to bottom rows are the intensity patterns of *I* = |*E_x_*|^2^, |*E_y_*|^2^ and |*E_x_*|^2^ + |*E_y_*|^2^. The insets are the enlarged views. The overlaid arrows show the schematic polarization states. The double arrows represent the linear polarization of illumination light.

Next, the single group of CSRs in the metasurface is extended to multiple groups of combined rings in the metasurface. The number of the groups of the designed metasurfaces is denoted by *N*. Specifically, we elaborate the design of metasurfaces based on *N* = 6, with semi-circular rings of zone integers *n*_2_ and *n*_1_ for right sets, and *n*_2_*’ = n*_2_ and *n*_1_*’ = n*_1_ for left sets are given in Table 1. Figure 4a shows the plot of the CSRs in the metasurfaces. Again, two blue semi-circular rings constitute a full ring with larger radius in a group, and two red ones constitute a full ring with smaller radius in each group. The solid blue and red semi-circular rings as the right set is extended to all the ring groups, of which all the right sets share the common right-shifted center *O_R_*, and the dotted blue and red rings represent the left sets of rings with the common left-shifted center *O_L_*. For a specific metasurface sample, the slits on the right sets of semicircular rings have the same rotation order *m_R_* and initial azimuth angle *φ*_0*R*_, while the slits on left sets of semicircular rings have the same rotation order *m_L_* and initial azimuth angle *φ*_0*L*_. Because the light field of the VB generated by all the *N* right sets of rings with the center, *O_R_* can be approximated as *U_hR_*(*O_R_’*) *≈ NU_h_*_1*R*_(*O_R_’*, *n*_2_, *n*_1_), and light field generated by the *N* left sets of rings with the center *O_L_* is *U_hL_*(*O_R_’*) *≈ NU_h_*_1*L*_(*O_L_’*, *n*_2_, *n*_1_), the light field on the observation plane produced by the *N* groups of CSRs of Fresnel zones is expressed as:

(17) $$\begin{array}{ll} U_{hc} & =U_{hR}(O_{R}{}^{\prime })+U_{hL}(O_{L}{}^{\prime })\\ & \approx NU_{h1R}(O_{R}{}^{\prime },\;n_{2},\;n_{1})+NU_{hL}(O_{L}{}^{\prime },\;n_{2}{}^{\prime },\;n_{1}{}^{\prime }) \end{array}$$

The above equation demonstrates that with the designed metasurface sample, two vortices or VBs are obtained at points *O_R_’* and *O_L_’* on the observation surface, depending on whether light of CP or linear polarization is used for the illumination. Seriously speaking, the approximation for *U_hR_*(*O_R_’*) may result in some error. For the Bessel function *J_l_*(*ar_f_*) in Equation (11) with *a = kr_n/_z*, the positions of the first maxima corresponding to different zones are different, and the superposition of Bessel functions will result in a widened doughnut for the formed vortex beam, which may be expressed by the confluence hypergeometric function [39]. However, the topological charge and the properties of the vortex beam remain unchanged. Therefore, the formed vortex beams with the widened doughnut can be roughly regarded as the Bessel vortex beams.

Figure 4b,c show the pattern and the enlarged view of metasurface sample with *m*_R_*= m_L_ =* 1*/*2, and *φ*_0*R*_ *= φ*_0*L*_ *=* 0°, respectively. Figure 4d shows FDTD intensity patterns of different VBs produced by the metasurface sample under illuminations of light linearly polarized in different directions. From left to right, the intensity patterns are for radially, 135°-slanted, azimuthally, and 45°-slanted polarized VBs, respectively, corresponding to the linear polarization direction of the illuminating light is in horizontal, 45°, vertical, and 135° directions, respectively. The patterns from top to bottom rows are, respectively, for the component intensities of *|E_x_|*^2^, *|E_y_|*^2^ and the total intensities of *I = |E_x_|*^2^*+|E_y_|*^2^ of the corresponding VBs. The inset intensity patterns in the upper left and upper right corners of each pattern are the enlarged view of the left-shifted and right-shifted VBs. The schematic arrows overlaid on the total light intensity patterns in the bottom row indicate the polarization states of the VBs. These results demonstrate that with the designed metasurface, the generation of dual VBs is well realized.

### 3.3. The FDTD Results for the Generated Dual Vector Beams of Different Orders and Polarization States

**Figure 5:**
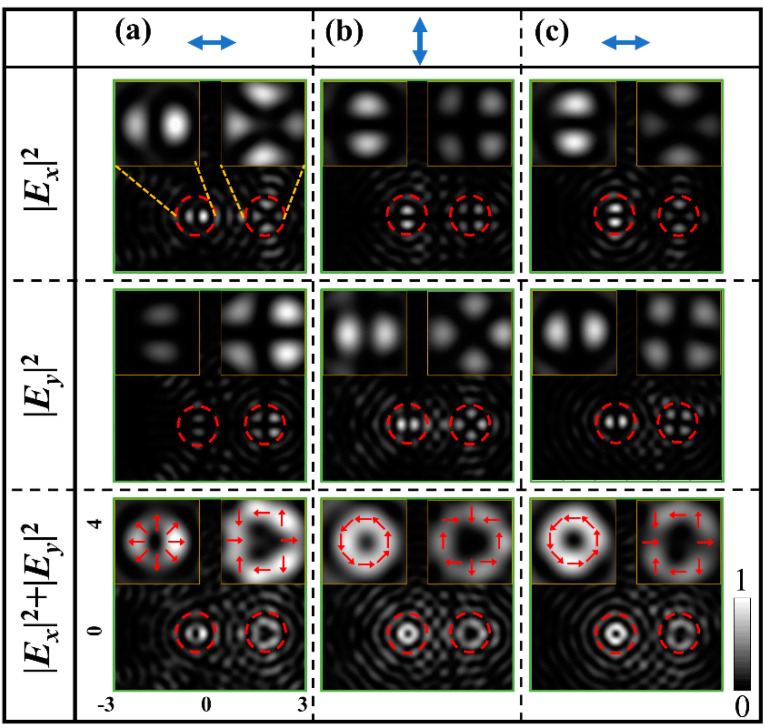
The intensity patterns of the dual VBs with different polarization states. (**a**) The radially polarized VBs with order *l_R_* = 2 and *l_L_* = 1. (**b**) The azimuthally polarized VBs with order *l_R_* = 2 and *l_L_* = 1. (**c**) The azimuthally polarized VBs with order *l_L_* = 1 and the radially polarized VBs with order *l_R_* = 2. From top to bottom, the intensity patterns are I = |*E_x_*|^2^, |*E_y_*|^2^ and |*E_x_*|^2^ + |*E_y_*|^2^ of the two separated VBs. The insets are the enlarged views. The overlaid arrows show the schematic polarization states. The double arrows represent the linear polarization of illumination light.

When the left- and right-shifted sets of the semicircular rings of the metasurface have different rotation orders, i.e., *m_R_ ≠ m_L_*, the metasurface can produce the left- and right-shifted VBs of different orders. We design the metasurface sample with *m_R_* = 1, *m_L_* = 1/2 and *φ_R_ = φ_L_ =* 0, leading to *l_R_* = 2 and *l_L_* = 1, which generates the second-order and first-order VBs at *O_R_’* and *O_L_’*, respectively. Under the illumination of horizontal linear polarization, this sample generate the radially polarized VBs of second- and first-orders, and intensity patterns of FDTD simulations are shown in Figure 5a, where from the top to bottom rows are the component intensities *|E_x_|*^2^, *|E_y_|*^2^ and the total intensity *I = |E_x_|*^2^ *+ |E_y_|*^2^, respectively. In Figure 5b, the results are shown for azimuthally polarized VBs of second- and first-orders by the same sample under illumination vertical linear polarization. In addition, we designed an additional metasurface sample with *m_R_* = 1, *m_L_* = 1/2, *φ_R_ = π/*4 and *φ_L_ =* 0 to demonstrate the case of *φ_R_* *≠* *φ_L_.* With this sample, the azimuthally polarized VB of the order *l_R_* = 2 and the radially polarized VB of *l_L_* = 1 are generated simultaneously at *O_R_’* and *O_L_’*, respectively, and Figure 5c shows the patterns of the component the total intensities of the sample with FDTD simulations.

## 4. Discussion

It can be seen from the above results that different dual VBs can be generated by adjusting the direction of linear polarization of the incident light. The basis is that when the direction of the incident linear polarization is at the angle of *ϕ*_0_ with respect to the horizontal direction, the terms of *E^σ^*^(1)^ and *E^σ^*^(−1)^ in Equation (12) are replaced by *E^σ^*^(1)^*e^iϕ^*^0^ and *E^σ^*^(−1)^*e*^-*iϕ*0^ with the phase factors introduced, respectively. Correspondingly, the first and the second terms on the right-hand side of Equation (14) are imposed to phase factors *e^iϕ^*^0^ and *e*^-*iϕ*0^, respectively, and thereby the polarization states of the dual VBs are modulated by (2*φ*_0_ *− ϕ*_0_). This demonstrates that the initial orientation of the slits and the linear polarization of the incident light jointly control the polarization states of VBs. Furthermore, according to Equation (16), by designing the initial orientation of the slits *φ*_0*R*_ on the set of right-shifted semicircular rings, *φ*_0*L*_ on the right sets of semicircular rings, and *φ*_0*L*_ on the left sets of semicircular rings, the dual VBs with different polarization states are generated at *O_R_’* and *O_L_’*.

**Figure 6:**
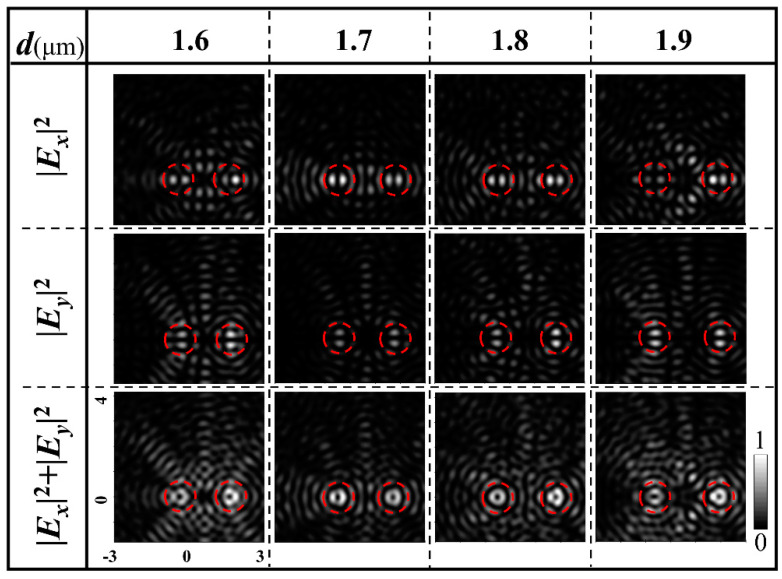
The evolution of intensity patterns of the two radially polarized dual VBs with the increase in distance *d* between the two beams.

In addition, the light field on the observation plane is the superposition of fields produced by the two sets of CSRs. Because the field of each set is approximated as a Bessel VB, at each center of the two beams, the interference intensity should be zero for yielding the VB of better quality. Though difficult in a serious perspective, it may be improved essentially by taking advantage of the positions of zero values of the Bessel functions, in the way that the center of one shifted VB coincides with the position of certain zero values of the Bessel function of the other beam. In practical performance, this is realized by properly optimizing the shifts *d_L_* and *d_R_* of the two sets of rings. To demonstrate the influence of the distance between the two VBs *d = d_L_ + d_R_* on the results, Figure 6 shows the patterns of the component and total intensities *|E_x_|*^2^, *|E_y_|*^2^ and *I = |E_x_|*^2^ *+ |E_y_|*^2^ by FDTD simulations when distance *d* takes the values 1.6, 1.7, 1.8, and 1.9 μm. All the parameters of the corresponding samples are the same as sample for Figure 4 except for *d*. It is seen that the VBs are optimized with the best beam petals when *d* takes 1.8 μm. However, there are also limitations if the metasurfaces are designed to generate vector beams of large orders. For the large-order vector beams, the doughnut diameters become very large, which require large shifts of the centers of the combined semicircular rings to avoid the crosstalk between the dual beams; this requires a large difference of the ring integers *n*_1_ and *n*_2_, leading to a lower utilization efficiency of the rings. To generate large-order vector beams of high quality and to have high utilization efficiency of the rings simultaneously, very sophisticated—and maybe, very time-consuming—calculations of the light wavefield for optimizing the nanostructures and shifts of combined ring centers are needed for selecting suitable *n*_1_ and *n*_2_.

The efficiency of the beam transformation can be calculated by the FDTD simulations. A single nanoslit at azimuth θ = 0 on the innermost ring with zone integer *n_1_* = 15 is taken for the calculation. With the used parameters, the single slit with dimension of 300 nm × 100 nm occupies the area of ΔA*_s_* = 1 μm × 0.361 μm, the orientation angle of slit with respect to the *x*-axis is set as *π*/8, and the slit element is illuminated with horizontal linearly polarized light. The ratio of the energy flow of the outgoing light to the energy flow of the incident light in ΔAs is calculated, and the conversion efficiency 3.49% is obtained. In fact, because there are no slits arranged in the central area with *n_1_* less than 15, the efficiency of the metasurface should be less than this value.

We note that it is possible to extend the metasurface to the generation of a 2D array of VBs. For a given array to be generated, the shifted center of each set of the CSRs corresponds to the position of each beam in the array. The zone integer *n*_1_ and *n*_2_ and the orientation of the combined rings are determined by the size and the direction of shift of center, i.e., by the position of the corresponding VB. In the design of a metasurface, all the CSRs are selected to ensure that their centers can shift positions of the corresponding beams without overlap of the rings.

Although in our work, only the theoretical principle and design method for metasurfaces of CSRs are focused on, they are demonstrated by results of the FDTD simulation. Here, we give some further analysis on the possible experimental measurements and demonstrations. Regarding to experimental performance, the designed metasurface sample can be directly fabricated by focusing ion beam etching (FIB) by directly using the nanostructures of the sample obtained from the results of FDTD simulations. The fabricated sample can be measured in an optical setup similar to that of [40]. The light from the He-Ne laser passes through a half-wave plate to adjust the linear polarization directions of the illuminating light. With linearly polarized light illuminating the sample, the dual VBs are obtained on the designed focal plane behind the sample. The patterns of the dual beams are magnified by an objective, and the polarization components of the dual beams is analyzed by a polarizer and recorded by an S-CMOS detector. Thus, measurements of the generated VBs and the verifications for the feasibility of the metasurface design may be realized.

## 5. Conclusions

By proposing the novel metasurface of CSRs with units of orthogonal slit pairs, the generation of dual VBs is realized. With a right-shifted set and a left-shifted set of the semicircular rings in a group of CSRs, two VBs are generated at the shifted center positions on the observation plane. The independently designed rotation order and initial orientation angle of slit units on each set, along with the adjustable linear polarization of illumination, flexibly control the right and left-shifted VBs with different orders and polarization states. Specifically, different metasurface samples are designed to generate two identical VBs of radial, azimuthal, and slanted polarizations of first order, to generate two VBs of radial polarization of first and second-order, and to generate VBs of radial polarization of first-order and azimuthal polarization of second-order. All the results validate the feasibility of the method. We expect the method may be extended to generate higher-order VBs in multiple channels, and we believe the work would be of importance in broadening the application area of VBs in miniature regimes.

## Figures and Tables

**Table 1:** Parameters for the design of metasurface samples with 6 groups of CSRs.

Group of Semicircular Rings		1	2	3	4	5	6
Solid blue line, *O_R_*	*n*_2_	19	23	27	31	35	39
Solid red line, *O_R_*	*n*_1_	15	19	23	27	31	35
Dotted blue line, *O_L_*	*n*_2_ *’*	19	23	27	31	35	39
Dotted bed line, *O_L_*	*n*_1_ *’*	15	19	23	27	31	35
*r_n_*_1_ (μm)		19.62	22.43	25.17	27.88	30.55	33.21
*r_n_*_2_ (μm)		16.73	19.62	22.43	25.17	27.88	30.55

*n*_2_ > *n*_1_, *n*_2_ *= n*_2_*’*, *n*_1_ *= n*_1_*’, d_L_ =* 0.53 μm, *d_R_ =* 1.80 μm.

## Data Availability

All data are contained within the article.
